# A comparison of dietary habits and sleep in adolescent female gymnasts with and without low energy availability

**DOI:** 10.1080/15502783.2025.2550155

**Published:** 2025-08-27

**Authors:** Hannah K. Eberhardt, Michael J. Ormsbee, Patrick G. Saracino

**Affiliations:** a University of South Carolina Upstate, Department of Human Performance and Health, Spartanburg, SC, USA; b Florida State University, Institute of Sports Sciences Medicine, Tallahassee, FL, USA; c University of KwaZulu-Natal, School of Health Sciences, Discipline of Biokinetics, Exercise and Leisure Sciences, Durban, South Africa

**Keywords:** Energy intake, sleep, Female Athlete, Macronutrients

## Abstract

**Background:**

This study aims to determine low energy availability prevalence (LEA) in adolescent female artistic gymnasts and to compare the dietary habits and sleep between those with LEA and those without.

**Methods:**

Height, weight, and body composition (BodPod) were measured following an overnight fast. Participants were then fitted with hip- and wrist-worn accelerometers to assess exercise energy expenditure (EEE) and sleep, respectively, for three consecutive days. Daily energy intake (EI) was assessed using a digital food log and evaluated against the International Society of Sports Nutrition (ISSN) dietary recommendations for athletic populations. As this was a free-living study, participants were asked to maintain their normal behaviors.

**Results:**

Twelve female artistic gymnasts (15 ± 1 yrs, 160.6 ± 5.5 cm, 55.8 ± 4.6 kg, 14.9 ± 6.4% body fat, 47.4 ± 4.3 kg fat-free mass (FFM)) participated in the study. Mean EA was 28.9 ± 13.5 kcals/kg^−1^FFM with six gymnasts (50%) presenting in a LEA state. Gymnasts with LEA consumed significantly fewer calories than those without (1451 ± 447 vs 2388 ± 458 kcals; *p* = 0.008). Those in a LEA state also had significantly lower protein (0.8 ± 0.2 vs 1.5 ± 0.4 g/kg; *p* = 0.007) and carbohydrate (3.1 ± 0.8 vs 4.8 ± 0.8 g/kg; *p* = 0.004) intake compared to gymnasts without LEA. Based on ISSN recommendations, 33.3% of gymnasts met PRO (1.4–2.0 g/kg^−1^), 8.3% met CHO (5–7 g/kg^−1^), while 100% met recommendations for fat (20–35% of total EI). EEE was 550 ± 131 kcal^−1^, with no differences by EA status. Those with LEA spent less time in bed (390 ± 56 vs 498 ± 35 min; *p* = 0.002) and had a lower total sleep time (346 ± 57 vs 439 ± 33 min; *p* = 0.006), yet fewer number of awakenings (17 ± 6 vs 24 ± 4; *p* = 0.035) compared to gymnasts without LEA. No differences in body composition or other sleep parameters were observed.

**Conclusions:**

In the present study, 50% of gymnasts presented in a LEA state. Regardless of EA status, most gymnasts did not meet the dietary recommendations for protein or carbohydrates. Additionally, those in a LEA state presented with sleep impairments, which could potentially impair recovery and performance. These findings underscore the need for future research to address the dietary and sleep habits in adolescent gymnasts to optimize health and athletic performance.

